# The accuracy and completeness for receipt of colorectal cancer care using Veterans Health Administration administrative data

**DOI:** 10.1186/s12913-016-1294-9

**Published:** 2016-02-11

**Authors:** Eric A. Sherer, Deborah A. Fisher, Jeffrey Barnd, George L. Jackson, Dawn Provenzale, David A. Haggstrom

**Affiliations:** Department of Chemical Engineering, Louisiana Tech University, Ruston, LA USA; Center for Health Information and Communication, Richard L. Roudebush Veterans Affairs Medical Center, Indianapolis, IN USA; Center for Health Services Research in Primary Care, Durham Veterans Affairs Medical Center, Durham, NC USA; Department of Medicine, Duke University Medical Center, Durham, NC USA; Duke Cancer Institute, Durham, NC USA; Department of Medicine, Indiana University School of Medicine, Indianapolis, IN USA; Center for Health Services Research, Regenstrief Institute, Inc., Indianapolis, IN USA; Indiana University Cancer Center, Indianapolis, IN USA

**Keywords:** Colorectal cancer, Cancer surveillance, Cancer treatment, Administrative data, Electronic medical record, Veterans Health Administration

## Abstract

**Background:**

The National Comprehensive Cancer Network and the American Society of Clinical Oncology have established guidelines for the treatment and surveillance of colorectal cancer (CRC), respectively. Considering these guidelines, an accurate and efficient method is needed to measure receipt of care.

**Methods:**

The accuracy and completeness of Veterans Health Administration (VA) administrative data were assessed by comparing them with data manually abstracted during the Colorectal Cancer Care Collaborative (C4) quality improvement initiative for 618 patients with stage I-III CRC.

**Results:**

The VA administrative data contained gender, marital, and birth information for all patients but race information was missing for 62.1 % of patients. The percent agreement for demographic variables ranged from 98.1–100 %. The kappa statistic for receipt of treatments ranged from 0.21 to 0.60 and there was a 96.9 % agreement for the date of surgical resection. The percentage of post-diagnosis surveillance events in C4 also in VA administrative data were 76.0 % for colonoscopy, 84.6 % for physician visit, and 26.3 % for carcinoembryonic antigen (CEA) test.

**Conclusions:**

VA administrative data are accurate and complete for non-race demographic variables, receipt of CRC treatment, colonoscopy, and physician visits; but alternative data sources may be necessary to capture patient race and receipt of CEA tests.

## Background

Approximately 3,400 colorectal cancers (CRC) are diagnosed in the Veterans Health Administration (VA) each year [1]. Considering CRC’s 5 year survival rate of 65.2 % [2], most of these veterans will become long-term survivors. As such, the receipt of CRC-related care, which includes curative intent treatment for CRC (i.e. surgical resection, chemotherapy, and radiation) [3, 4] as well as tests for assessment and surveillance after the diagnosis of CRC (i.e. colonoscopy, physician visit, and carcinoembryonic antigen (CEA) test) [5], is of broad interest for both veteran and non-veteran populations [6–9].

An accurate and efficient method is needed to measure receipt of CRC-related care. Potential data sources include surveys [10], health record review [11], and health claims (billing) data [10, 12–17]. The VA uses an electronic health record system, known as the Computerized Patient Record System (CPRS), which allows searching for procedure notes and other services within a given patient record to determine what care has been provided [18]. However, review of health records for research purposes typically occurs on a facility by facility basis because patient health records are not routinely shared between medical centers except for direct patient care. Use of health claims data has practical advantages in terms of access to large samples across multiple sites and low incremental costs. Thus, in this methodologic study, our objective was to compare the receipt of CRC-related care in VA according to administrative data compared with data from a manual chart abstraction of the electronic health record performed during a quality improvement collaborative (EHR/QI) called the Colorectal Cancer Care Collaborative (C4). C4 was undertaken to improve processes of care delivery for CRC [7, 19].

Administrative data has been compared with health record review for receipt of cancer surveillance tests [12] and colonoscopy [10] by Medicare patients; receipt of colonoscopy prior to CRC diagnosis [17] and CRC surgery [16] in Alberta, Canada; and receipt of colonoscopy, surgery, chemotherapy, and radiation by CRC patients in New South Wales, Australia [15]. But this study is the first to attempt to validate the use of VA administrative data for measuring the receipt of CRC treatment and surveillance care. The objectives of this study are to identify types of care in which the use of VA administrative data to measure receipt of CRC-related care is reasonably complete and accurate as well as measures in which the VA administrative data may not be sufficiently complete or accurate so alternative methods may be necessary to collect this data in studies involving VA data.

## Methods

The study was approved by the Institutional Review Board at Indiana University (0805–61), the VA research committee at the Roudebush VA Medical Center, and the Institutional Review Board at Louisiana Tech University (HUC 1048). The research is in compliance with the Helsinki Declaration.

### Data sources

C4 was a national quality improvement project involving teams from 28 VA medical centers (VAMCs) with at least one team from each of the 21 regional Veterans Integrated Service Networks (VISNs) covering the entire U.S. (see Jackson et al., 2013 [9] for a detailed description of the C4 manual data abstraction.). Briefly, C4 sites used local cancer registries or clinical patient lists to identify a cohort of newly diagnosed CRC patients. C4 sites used an abstraction tool, called the Cancer Care Quality Measurement System (CCQMS), to systematically guide retrospective manual abstraction of approximately 230 data elements from the VA’s Computerized Patient Record System (CPRS). These elements were used to evaluate compliance with 25 quality indicators, based upon National Comprehensive Cancer Network (NCCN) guidelines, for colon and rectal cancer treatment. These quality indicators include receipt of curative intent CRC treatment and dates of CRC-related tests received by individual patients (which the current study uses to quantify the accuracy and completeness of VA administrative data for measuring the receipt of this care). Abstractors were cancer registrars, clinical, or administrative staff. The level of training and number of abstractors varied between sites but data variability was limited by a standard computerized data abstraction tool, data definition documents, availability of a centralized help desk, and training of abstractors [9]. A validation of inter-observer health record abstraction accuracy was not performed for the C4 study, but its data abstractors reported a high level of confidence in knowing what data needed to be entered, where to find the data in medical records, and that the instructions were clear [9].

### Study population

The study cohort included patients identified during C4 with an incident diagnosis of non-metastatic (AJCC collaborative stage I, II, or III) CRC between January 1, 2004 and October 1, 2006 with complete data collection in which all NCCN quality measures and surveillance tests were evaluated by C4 abstractors. Follow-up care data were collected by October 1, 2007. But, because there was also no documentation of when each manual search of the EHR/QI was conducted, we assumed that the final search for a patient was conducted on the last documented date in the EHR/QI data for that patient. Patients with stage IV CRC were excluded from the current study because these patients may not be eligible for curative intent treatment and the American Society of Clinical Oncology (ASCO) guidelines for CRC surveillance care are more uniformly defined for patients diagnosed with stage I-III CRC [5].

### Electronic health record / quality improvement (EHR/QI) data

While all C4 study patients were eligible for data collection for at least 12 months after CRC diagnosis, the EHR/QI data collection was non-systematic. Data abstractions were not done at a fixed period of time after diagnosis. Therefore, a patient’s data were censored at the latest date recorded by the EHR/QI abstractor so only the period of care reviewed by the abstractors was used for analysis.

### Administrative data

VA administrative data were drawn from inpatient (acute, extended, observation, and non-VA care) and outpatient files from January 1, 2004 through October 1, 2007. This period spans the EHR/QI data collection from the earliest diagnosis of CRC in EHR/QI to one year after the latest diagnosis of CRC when EHR/QI surveillance data collection ended. For the inpatient file, the main, procedure, and surgery datasets were included. CRC-related care events were identified using relevant Current Procedure Terminology (CPT-4) procedure codes and International Classification of Diseases, 9^th^ revision, Clinical Modification (ICD-9-CM) diagnosis codes (Table 1). The ICD-9 codes are used in both inpatient and outpatient administrative files and CPT-4 codes are used in the outpatient administrative data files so both types of codes are required to identify all diagnoses and procedures. VA administrative data are based on information entered into CPRS and coded (e.g., ICD-9 and CPT-4 codes) at the time of the patient encounter. VA administrative data are then stored in a centralized location from across the VA healthcare system. We obtained the VA administrative data for this study by querying the VA national data system (NDS) for records containing any of the codes listed in Table 1 during the study period for the study patients. Demographic variables were identified from the visit dataset in the outpatient file. In addition, demographic characteristics were also drawn from the VA Central Cancer Registry (VACCR) for a separate comparison with the EHR/QI data.

**Table 1:** ICD-9 diagnostic, CPT-4 procedure, and laboratory codes used to identify CRC-related treatments and test events

Test or Treatment	ICD-9 diagnostic code	CPT-4 procedure code
Colonoscopy	45.22, 45.23, 45.25, 45.41, 45.42, 45.43, 48.36	44,388, 44,389, 44,392, 44,393, 44,394,45,378, 45,380, 45,382, 45,383, 45,384, 45,385, 45,391, G0105
Carcinoembryonic antigen (CEA)	795.81	82,378
Physician visit		99201-99205, 99211-99215, 99241-99245, 99381-99387, 99391-97
Surgical resection^a^	45.7, 45.71-45.79, 45.8, 45.81- 45.89, 48.4, 48.41-48.49, 48.5, 48.51-48.59, 48.6 48.61-48.69	44140, 44141, 44143-44160, 44024-44213
Chemotherapy	99.25, V58.1, V66.2, V67.2, E930.7, E933.1	36260, 36640, 96400-96425
Radiation	92.2, 92.21-92.26,92.29; 92.3-92.39, V58.0, V66.1, V67.1	77260-77525

^a^Cooper et al., 2000 [33] identified the ICD-9 and CPT-4 codes associated with the 4 levels of surgery documented in SEER: biopsy, local excision, bypass surgery, and resection. Only the codes associated with “resection” were used

### Variables collected

CRC-related care included both curative intent treatments and surveillance tests done after CRC diagnosis to detect CRC recurrence. Receipt of the following curative intent treatments were drawn from the EHR/QI and VA administrative data: surgical resection, neoadjuvant chemotherapy, and adjuvant chemotherapy, and neoadjuvant radiation, and adjuvant radiation therapies. In VA administrative data, chemotherapy or radiation prior to the date of surgical resection in EHR/QI data were considered neoadjuvant treatment and chemotherapy or radiation after this date was considered adjuvant treatment; the completion of treatment courses was not evaluated.

Receipt of the following surveillance tests [20] were compared between the EHR/QI and VA administrative data: colonoscopy [21], CEA test [5], and physician visits. Two administrative records were considered to be the same event if both events 1) were of the same type and 2) occurred within a specified time window of each other. Colonoscopies and surgeries that occurred within 30 days of each other were considered the same event and CEA tests and physician visits that occurred within 7 days of each other were considered the same event [22]. In these cases, the event that occurred first was considered the “true” event because, for a short time window, the repeat test is likely a completion or follow-up to the first test and not an independent event. These time windows were applied to every instance of every test.

The demographic variables of date of birth, date of death, gender, race, and marital status were collected from EHR/QI and VA administrative data as well as the VACCR.

### Analysis

Patients in the VA administrative data were matched with patients in the EHR/QI by their social security number. For demographic variables, values from VA administrative data were compared to EHR/QI data for accuracy and completeness (see Table 2). The accuracy of a variable was assessed using the percent agreement between the values in EHR/QI and VA administrative data for patients who have non-missing observations in both sources. The completeness of a variable was the percentage of patients with an observation in the EHR/QI data who also had the observation in VA administrative data. While this metric misses EHR/QI false negative tests, it does demonstrate the ability of the VA administrative data to identify outcomes that would also be identified by manual abstraction.

**Table 2:** Metrics used to evaluate the accuracy of the VA administrative data relative to EHR/QI manual abstraction data

Variable	Accuracy
Demographics	Percent agreement
Post-diagnosis treatment events	Positive predictive value
Receipt of type of care at any time while eligible	Kappa statistic

To evaluate the receipt of individual surveillance test events, the accuracy of VA administrative data relative to the EHR/QI data for events of each type of post-diagnosis test was assessed using the positive predictive value relative to the EHR/QI data. The advantage of the positive predictive value is that it assesses the overall receipt of a test and not individual test events; this is important because it was unclear whether the EHR/QI continued to collect surveillance events once the recommended surveillance was achieved. The completeness of VA administrative data for events of each type of post-diagnosis test was the fraction of test events in the EHR/QI also present in VA administrative data.

To evaluate whether a type of care (curative treatment or surveillance test) was received at any time during the eligible time period, the accuracy of VA administrative data were determined using kappa statistics for agreement versus the EHR/QI data. The kappa statistics for agreement for each type of care was based on comparing the EHR/QI and VA administrative data predicted receipt of care for individual patients over the eligible time interval. In general, kappa values <0 indicate less than chance agreement, 0.01–0.20 indicate slight agreement, 0.21–0.40 indicate fair agreement, 0.41–0.60 indicate moderate agreement, 0.61–0.80 indicate substantial agreement, and 0.81–1.00 indicate almost perfect agreement [23]. The sensitivity for identifying whether a type of CRC-related care was received at any time during the eligible time period was the fraction of patients documented by the EHR/QI data as receiving a type of care who also had documentation of that care in VA administrative data.

All analyses were performed using R 2.15.2.

## Results

The EHR/QI project completed chart abstraction for 624 patients with AJCC collaborative stage I, II, or III CRC; as shown in Table 3, the demographic characteristics of the EHR/QI cohort are similar to those of all patients diagnosed with AJCC collaborative stage I-III CRC in the VACCR during the study period. A total of 618 patients (99.0 %) identified in the EHR/QI process were also identified in VA administrative data. A total of 413 of these patients were also present in the VACCR with AJCC collaborative stage I, II, or III cancer.

**Table 3:** Patient characteristics and agreement between VA administrative data and EHR/QI chart abstraction

Characteristic	EHR/QI (*N* = 624)	All stage I-III VACCR (*N* = 4,870)	*p*-value	VA administrative data (*N* = 618)	EHR/QI vs. VA administrative data	VA Central Cancer Registry (*N* = 413)	EHR/QI vs. VA Central Cancer Registry
Demographic							
Age at diagnosis – median (q1, q3)	69.0 years (60.3, 78.1)	69 years (60,77)	0.64	N/A	N/A	69 years (60, 78)	98.3 % (406 / 413)
Date of birth - % (n / N)					98.7 % (610 / 618)		N/A
Date of death - % (n / N)					98.1 % (606 / 618)		N/A
Gender - % (n / N)			0.84		100 % (618 / 618)		100 % (413 / 413)
Male	98.1 % (612 / 624)	97.9 % (4,768 / 4,870)		98.2 % (607 / 618)		99.0 % (409 / 413)	
Female	1.9 % (12 / 624)	2.1 % (102 / 4,870)		1.8 % (11 / 618)		1.0 % (4 / 413)	
Unknown	0.0 % (0 / 624)	0.0 % (0 / 4,870)		0.0 % (0 / 618)		0.0 % (0 / 413)	
Race - % (n / N)			0.27		93.0 % (213 / 229)		98.5 % (392 / 398)
White	80.6 % (503 / 624)	81.8 % (3,986 / 4,870)		30.6 % (189 / 618)		80.1 % (331 / 413)	
Black	12.8 % (80 / 624)	15.2 % (739 / 4,870)		7.0 % (43 / 618)		14.8 % (61 / 413)	
Other	4.2 % (26 / 624)	1.0 % (24 / 4,870)		0.3 % (2 / 618)		2.9 % (12 / 413)	
Unknown	2.4 % (15 / 624)	2.1 % (100 / 4,870)		62.1 % (384 / 618)		2.2 % (9 / 413)	
Marital status - % (n / N)			0.73		91.4 % (512 / 560)		95.2 % (357 / 375)
Single	39.9 % (249 / 624)	39.1 % (1,905 / 4,870)		51.6 % (319 / 618)		48.9 % (202 / 413)	
Married	50.8 % (317 / 624)	51.7 % (2,517 / 4,870)		48.4 % (299 / 618)		51.1 % (211 / 413)	
Unknown	9.3 % (58 / 624)	9.2 % (448 / 4,870)		0.0 % (0 / 618)		0.0 % (0 / 413)	
Cancer							
Location			0.45		N/A		97.0 % (392 / 404)
Colon	69.7 % (435 / 624)	71.1 % (3,465 / 4,870)		N/A		74.6 % (308 / 413)	
Rectum	28.2 % (176 / 624)	26.7 % (1,298 / 4,870)		N/A		25.4 % (105 / 413)	
Unknown	2.1 % (13 / 624)	2.2 % (107 / 4,870)		N/A		0 % (0 / 413)	
AJCC collaborative stage			0.70		N/A		95.2 % (393 / 413)
I	30.0 % (187 / 624)	31.0 % (1,511 / 4,870)		N/A		30.5 % (126 / 413)	
II	37.0 % (231/624)	36.2 % (1,763 / 4,870)		N/A		36.8 % (152 / 413)	
III	33.0 % (206 / 624)	32.7 % (1,596 / 4,870)		N/A		32.7 % (135 / 413)	
Curative treatment - % (n / N)							
Surgical resection	95.8 % (598 / 624)	N/A		91.7 % (567 / 618)	92.7 % (573 / 618)	N/A	N/A
Date of Surgery		N/A			96.9 % (540 / 557)	N/A	N/A
Neoadjuvant chemotherapy	13.0 % (81 / 624)	N/A		5.8 % (36 / 618)	91.7 % (567 / 618)	N/A	N/A
Neoadjuvant radiation	13.6 % (85 / 624)	N/A		7.6 % (47 / 618)	92.9 % (574 / 618)	N/A	N/A
Adjuvant chemotherapy	27.1 % (169 / 624)	N/A		30.1 % (186 / 618)	81.6 % (504 / 618)	N/A	N/A
Adjuvant radiation	5.6 % (35 / 624)	N/A		21.8 % (135 / 618)	80.3 % (496 / 618)	N/A	N/A

According to the EHR/QI data abstraction, the average age at the diagnosis date was 69.0 years and the population was predominantly male (98.1 %) and white (80.6 %) as shown in Table 3. Roughly half (50.8 %) of the patients were married. CRC cases were fairly evenly distributed between stage I (30.0 %), II (37.1 %), and III (33.0 %) and the majority of patients had colon cancer (69.7 %). Most patients had a surgical procedure (95.8 %; 598/624) and some received neoadjuvant chemotherapy (13.0 %; 81/624), adjuvant chemotherapy (27.1 %; 169/624), neoadjuvant radiation (13.6 %; 85/624), or adjuvant radiation (5.6 %; 35/624). Post-diagnosis care was monitored for an average of 10.4 months (7.9 month standard deviation).

### Percent agreement and completeness of demographic characteristics

For the 618 of 624 patients in EHR/QI data also in the VA administrative data, the agreement between the EHR/QI and VA administrative data for demographic and CRC variables was 98.7 % (610/618) for date of birth, 98.1 % (606/618) for date of death, 100.0 % (618/618) for gender, 93.0 % (213/229) for race, and 91.4 % (512/560) for marital status (Table 3). VA administrative data contained complete data for all patients (100 %, 618/618) for date of birth, gender, and marital status. However, the VA administrative data contained patient data for race for only 37.9 % (234/618) of patients.

### Percent agreement and completeness of cancer and clinical characteristics

For the 413 EHR/QI patients also present in the VACCR with stage I, II, or III cancer, the CRC stage in the VACCR agreed with the EHR/QI data for 95.2 % (393/413) of patients and the agreement for CRC location was 97.0 % (392/407 with missing data for 6 EHR/QI patients). The agreement for number of lymph nodes examined and number of lymph nodes positive for cancer were 94.3 % (367/389 with missing data in EHR/QI for 19 patients and VACCR for 5 patients) and 98.2 % (385/392 with missing data in EHR/QI for 19 patients and VACCR for 8 patients), respectively. The agreement between the C4 and the VACCR for demographic and CRC variables was 98.3 % (406/413) for age at diagnosis, 100.0 % (413/413) for gender, 98.5 % (392/398) for race, and 95.2 % (357/375) for marital status. The VACCR contained complete patient data (100 %, 413/413) for age at diagnosis, gender, and marital status and race data for 96.3 % (398/413) of patients.

### Completeness and positive predictive value of VA administrative data for post-diagnosis test events

VA administrative data identified 174 of 229 (76.0 %; 95 % CI, 70.1–81.1 %) post-diagnosis colonoscopies, 294 of 1,117 (26.3 %; 95 % CI, 23.8–29.0 %) CEA tests, and 2,027 of 2,396 (84.6 %; 95 % CI, 83.1–86.0 %) physician visit events in the EHR/QI data (Table 4).

**Table 4:** Completeness and PPV for post-diagnosis test events in VA administrative data versus EHR/QI manual data abstraction

Test	Completeness (95 % CI)	Positive predictive value^a^ (95 % CI)	EHR/QI and VA administrative data	Only EHR/QI data	Only VA administrative data
Colonoscopy	0.760 (0.701, 0.811)	0.611 (0.551, 0.667)	174	55	111
Carcinoembryonic antigen	0.263 (0.238, 0.290)	0.541 (0.498, 0.584)	294	823	249
Physician visit	0.846 (0.831, 0.860)	0.319 (0.307, 0.330)	2,027	369	4,334

^a^A lower positive predictive value indicated that more events were identified in the administrative data that were not also identified by C4

The positive predictive value of VA administrative data for post-diagnosis test events identified in the EHR/QI data was 61.1 % (95 % CI, 55.1–66.7 %) for colonoscopy, 54.1 % (95 % CI, 49.8–58.4 %) for CEA tests, and 31.9 % (95 % CI, 30.7–33.0 %) for physician visits (Table 4). A lower positive predictive value indicated that more events were identified in the administrative data that were not also identified by C4.

### Sensitivity and agreement for receipt of CRC-related care (treatments and tests) at any time during eligible period

For treatments, VA administrative data identified 557 of 592 (94.1 %; 95 % CI, 91.8–95.6 %) patients who received surgical resection in the EHR/QI data; and the date of surgical resection agreed between the two data sources for 540 of these 557 (96.9 %; 95 % CI, 95.2–98.1 %) patients. VA administrative data identified 33 of 36 (91.7 %; 95 % CI, 76.4–97.8 %) patients who received neoadjuvant chemotherapy, 121 of 187 (65.1 %; 95 % CI, 57.7–71.8 %) who received adjuvant chemotherapy, 44 of 85 (51.8 %; 95 % CI, 40.7–62.6 %) who received neoadjuvant radiation, and 24 of 35 (68.6 %; 95 % CI, 50.6–82.6 %) who received adjuvant radiation (Table 5). VA administrative data and the EHR/QI data showed moderate agreement for receipt of neoadjuvant radiation (κ = 0.60; 95 % CI, 0.49–0.71), adjuvant chemotherapy (κ = 0.55; 95 % CI, 0.48–0.63), and neoadjuvant chemotherapy (κ = 0.53; 95 % CI, 0.40–0.65). The agreement was fair for the receipt of surgical resection (κ = 0.38; 95 % CI, 0.21–0.56) and adjuvant radiation (κ = 0.21; 95 % CI, 0.09–0.34).

**Table 5:** Sensitivity and kappa static for receipt of any CRC-related care in VA administrative data versus EHR/QI manual data abstraction

Test or Treatment	Sensitivity (95 % CI)	Kappa statistic (95 % CI)	EHR/QI and VA administrative data	Only EHR/QI data	Only VA administrative data	Neither data source
Colonoscopy	0.855 (0.797, 0.899)	0.66 (0.59, 0.72)	171	29	69	349
Carcinoembryonic antigen	0.497 (0.446, 0.549)	0.35 (0.28, 0.42)	190	192	24	212
Physician visit	0.996 (0.984, 0.999)	0.83 (0.78, 0.89)	498	2	27	91
Surgical resection	0.941 (0.918, 0.956)	0.38 (0.21, 0.56)	557	35	10	16
Neo-adjuvant chemotherapy	0.917 (0.764, 0.978)	0.53 (0.40, 0.65)	33	3	48	534
Adjuvant chemotherapy	0.651 (0.577, 0.718)	0.55 (0.48, 0.63)	121	65	48	383
Neo-adjuvant radiation	0.518 (0.407, 0.626)	0.60 (0.49, 0.71)	44	41	3	530
Adjuvant radiation	0.686 (0.506, 0.826)	0.21 (0.09, 0.34)	24	11	111	472

For post-diagnosis tests, VA administrative data identified 171 of 200 (85.5 %; 95 % CI, 79.7–89.9 %) patients who received a colonoscopy in the EHR/QI data, 190 of 382 (49.7 %; 95 % CI, 44.6–54.9 %) who received a CEA, and 498 of 500 (99.6 %; 95 % CI, 98.4–99.9 %) who received a physician visit. VA administrative data and the EHR/QI showed almost perfect agreement for whether there was a primary care visit (κ = 0.83; 95 % CI, 0.78–0.89) at any time during the eligible period. The agreement for colonoscopy was moderate (κ = 0.66; 95 % CI, 0.59–0.72) and the agreement for CEA tests was fair (κ = 0.35; 95 % CI, 0.28–0.42).

The supplemental accuracy measures of the specificity, positive predictive value, and negative predictive value of receipt of a type of care during the eligible period for VA administrative data versus the EHR/QI data are provided in Table 6.

**Table 6:** Specificity, PPV, and NPV for receipt of any CRC-related care in VA administrative data versus EMR/QI manual data abstraction

Test or Treatment	Specificity (95 % CI)	Positive Predictive Value (95 % CI)	Negative Predictive Value (95 % CI)
Colonoscopy	0.834 (0.795, 0.869)	0.713 (0.650, 0.768)	0.923 (0.890, 0.947)
Carcinoembryonic antigen	0.898 (0.851, 0.932)	0.888 (0.836, 0.925)	0.524 (0.475, 0.574)
History and physical	0.771 (0.682, 0.841)	0.949 (0.925, 0.965)	0.978 (0.917, 0.996)
Surgery	0.615 (0.407, 0.791)	0.982 (0.967, 0.991)	0.314 (0.195, 0.460)
Neo-adjuvant chemotherapy	0.918 (0.891, 0.938)	0.407 (0.301, 0.522)	0.994 (0.982, 0.999)
Adjuvant chemotherapy	0.889 (0.854, 0.916)	0.716 (0.641, 0.781)	0.855 (0.818, 0.886)
Neo-adjuvant radiation	0.994 (0.982, 0.999)	0.936 (0.814, 0.983)	0.928 (0.903, 0.947)
Adjuvant radiation	0.810 (0.775, 0.840)	0.178 (0.119, 0.255)	0.977 (0.958, 0.988)

## Discussion

The use of cancer registries linked with administrative data represents a widely accepted methodologic approach in cancer health services research to identify cancer treatment and surveillance care that may not be captured by tumor registries alone. Linked SEER-Medicare databases have been used to develop relapse algorithms [24]; evaluate receipt of CRC surgical resection [25], chemotherapy [26], and radiation [27]; and study cancer screening rates [28]. Similarly, linkages between VA cancer registries and VA administrative data could be used to evaluate care received within the VA system if this care is adequately captured in administrative data. The methodologic study presented here evaluated the completeness and accuracy of VA administrative data for CRC-related care by comparing procedures identified in the administrative data with those from a manual abstraction of electronic health records performed during the course of a quality improvement collaborative.

There was good agreement between VA administrative and EHR/QI data for demographic variables (91.4 % to 100 % agreement) and CRC treatments received (80.3 % to 92.9 % agreement). The agreements for treatment are similar to what has been observed in Medicare vs. record review for receipt of chemotherapy (88 % colon and 91 % rectal) [26] and radiation (93.5 %) [27]. As previously noted by others [29, 30], there is considerable missingness in VA administrative data for race (62.1 % missing). Our result is consistent with what is known about race in VA administrative data; according to the VIReC Technical Report: VA Race Data Quality, race is missing from 33–37 % of VA administrative data records for 2004–2005, although the quality of the data has been improving with time [31]. The missingness of race in the VA cancer registry was considerably lower (2.2 %) and may be considered as the primary source of race for studies involving VA patients with cancer.

VA administrative data were reasonably complete for post-diagnosis colonoscopy (76 %) and physician visit (84.6 %) events but the completeness for the CEA (26.3 %) events was considerable lower. This lower completeness for the CEA tests may be due to the coding of many of these procedures in laboratory data, which was not considered here, or a lower rate of coding laboratory tests in the administrative data. The agreement for receipt of colonoscopy (κ = 0.66) was moderate but lower than the almost perfect agreement that has been observed between Medicare data and health record review (κ = 0.89) [10]. But, as discussed below, the majority of the disagreements in VA data were due to colonoscopies in the VA administrative data that were not documented by the EHR/QI.

There were a significant number of procedures of all types identified in VA administrative data but not by the EHR/QI data manual abstraction process. For example, the positive predictive value for colonoscopy after diagnosis was 61.1 %, or 111 of the 285 colonoscopy events in VA administrative data were not in the EHR/QI data. This difference between administrative claims data and chart abstraction can be explained by either “false-positive” claims or incomplete/inaccurate abstraction. The latter explanation is the more compelling. Specifically, it was unclear whether the EHR/QI data continued to collect surveillance events once the recommended surveillance was achieved; this would lead to apparent false positives in the VA administrative data which would lower the positive predictive value. Also, differences between administrative data and manual abstraction may occur if abstractors misinterpreted coding directions (e.g., difficulty in defining a physician visit was noted as a limitation of the EHR/QI [9]), did not collect all procedures (e.g., local site variations [32] or patient met the indicator criteria so no additional procedures were collected), or simply missed procedures. Because the C4 was undertaken as part of a quality improvement effort rather than a systematic CRC surveillance study, these abstraction errors are likely to be more prevalent in the C4 data than traditional research studies. Previous studies stemming from the C4 have identified the greatest opportunity for improvement as timely surveillance of cancer survivors, however due to missing data, previous studies may have overestimated the size of the quality gap [7, 9]. Also of note is that colon and rectal cancer were not differentiated using VA administrative data which – because, in the EHR/QI, there are indicator variables for neoadjuvant chemotherapy and neoadjuvant radiation of rectal but not colon cancer as well as no indicator variable for adjuvant radiation [9] – likely led to many of the “false-positives” for these variables in VA administrative data.

There are several potential limitations to this study. While the receipt of CRC surveillance procedures was compared, the indication for the procedure (i.e. whether the procedure was for surveillance or diagnostic) was unknown. Without the indication to identify whether procedures were related, time windows were used to identify related procedures but this may combine two unrelated procedures together. There was also no documentation of when each manual search of the EHR/QI was conducted. We assumed that the final search for a patient was conducted on the last documented date in the EHR/QI data for that patient, but it is possible that subsequent searches were conducted that found no new information. We also assumed that all EHR/QI variables were searched for during each manual abstraction, but it is possible that not all variables were searched for during each examination of the health record. Of note, our study represents a comparison of administrative data and electronic health records in the VA system. While these methods may serve as a guide, they should be validated in other healthcare systems. In addition, the VA CRC patient population is predominantly male and diagnosed at an earlier stage than the general population [1] so the study cohort may not be generalizable. Finally, the comparison on patient characteristics in Table 3 was based patients whose data was not missing so there is the potential for selection bias.

## Conclusions

In summary, national Veterans Affairs administrative data can be a powerful tool to assess the quality of CRC surveillance care. However, before measures of care quality can be instituted, the accuracy of these data should be examined. Results from this study suggest that VA administrative data are accurate and complete for non-race demographic variables, receipt of CRC treatment, colonoscopy, and physician visits and may be a potential alternative to manual abstraction for the measurement of CRC care. However alternative data sources will be necessary to capture patient race and receipt of CEA tests.

## Abbreviations

AJCC: American Joint Committee on Cancer
ASCO: American Society of Clinical Oncology
C4: Colorectal Cancer Care Collaborative
CCQMS: Cancer Care Quality Measurement System
CEA: carcinoembryonic antigen
CPRS: Computerized Patient Record System
CPT-4: Current Procedure Terminology
CRC: colorectal cancer
EHR/QI: electronic health record / quality improvement collaborative
ICD-9: International Classification of Diseases, 9^th^ revision
NCCN: National Comprehensive Cancer Network
NDS: national data system
SEER: Surveillance, Epidemiology, and End Results
VA: Veterans Health Administration
VACCR: VA Central Cancer Registry
VAMC: VA medical center
VIReC: VA Information Resource Center
VISN: Veterans Integrated Service Network
